# Gait Change Is Associated with Cognitive Outcome after an Acute Ischemic Stroke

**DOI:** 10.3389/fnagi.2017.00153

**Published:** 2017-05-18

**Authors:** Sharmila Sagnier, Pauline Renou, Stéphane Olindo, Sabrina Debruxelles, Mathilde Poli, François Rouanet, Fanny Munsch, Thomas Tourdias, Igor Sibon

**Affiliations:** ^1^CHU Pasteur 2, Unité Neuro-VasculaireNice, France; ^2^UMR 5287 Centre National de la Recherche Scientifique, Université de Bordeaux, EPHE PSL Research UniversityBordeaux, France; ^3^CHU de Bordeaux, Unité Neuro-VasculaireBordeaux, France; ^4^CHU de Bordeaux, Neuroradiologie Diagnostique et ThérapeutiqueBordeaux, France; ^5^Health and Human Sciences Department, Université de BordeauxBordeaux, France

**Keywords:** ischemic stroke, gait, cognitive impairment, prognosis, longitudinal study

## Abstract

**Background:** Cognition and gait have often been studied separately after stroke whereas it has been suggested that these two domains could interact through a cognitive-motor interference.

**Objective:** To evaluate the influence of gait changes on cognitive outcome after an ischemic stroke (IS).

**Methods:** We conducted a prospective and monocentric study including patients admitted for an acute supratentorial IS with a National Institute of Health Stroke Score ≤ 15. Cognition, gait and motor disability were evaluated at baseline, 3 months and 1 year post-stroke, using the Montreal Cognitive Assessment (MoCA), the 10-m walking test (10-MWT) and the Fugl-Meyer motor assessment (FMMA). The effect of changes in 10-MWT over the year of follow-up on MoCA changes was estimated using a generalized linear mixed model with FMMA, age and gender as covariates.

**Results:** Two hundred and Twelve patients were included (71% male, age 64 ± 13 years old). 10-MWT improved from baseline to 1 year (*p* < 0.001), as did MoCA (*p* < 0.001) and FMMA (*p* < 0.001) scores. Ninety-nine patients (47%) had a MoCA <26 at 1 year. Changes in 10-MWT were independently associated with changes in MoCA (β = −0.2, 95% CI −0.24 to −0.07, Bonferroni-corrected *p*-value = 0.002). Analyses of MoCA sub-scores suggested that changes in gait performance was associated with changes in executive functions and recall.

**Conclusion:** Gait performance is associated with cognitive outcome after a mild to moderate IS, suggesting that they should be managed together to improve post-stroke independence.

## Introduction

Cognitive and walking impairment are two major sources of post-stroke disability. Although most of the stroke survivors experience some degrees of recovery in walking within the first months following symptom onset, gait disturbances can persist and worsen functional outcome (Baetens et al., 2013). The burden of cognitive and gait impairment has often been studied separately while the cognitive effort required to detect environmental changes and to compensate postural perturbations when walking suggests that these two domains should be evaluated together (Montero-Odasso et al., 2012). The strong interaction between cognition and gait has been well-described in neurodegenerative disorders. Indeed, slowing gait has been observed among patients with mild cognitive impairment (Montero-Odasso et al., 2012), while cognitive impairment could worsen motor abilities among patients with parkinsonism (Amboni et al., 2012). More recently, this cognitive-motor interference has been evaluated in post-stroke studies (Haggard et al., 2000; Cockburn et al., 2003; Chen et al., 2013), suggesting both concurrent gait and cognitive worsening in dual tasks. A trend for the functional benefit of cognitive-motor training on gait performance and dual task performance following stroke has been observed in some studies, reinforcing the clinical relevance of a combined evaluation of cognition and gait (Montero-Odasso et al., 2012). However, most of these studies included small samples of patients, did not consider the dynamic process of post-stroke cognitive evolution and used specific tasks with high cognitive effort, which are difficult to apply in clinical practice.

The aim of the present study was to evaluate the relationship between changes in gait performance assessed by walking speed and cognitive outcome, evaluated by the Montreal Cognitive Assessment scale (MoCA; 7) over a 1-year follow-up period in a large sample of patients suffering from a recent ischemic stroke.

## Materials and methods

### Inclusion/exclusion criteria

Patients were recruited prospectively in a single center, the Bordeaux University Hospital, from June 2012 to February 2015. Inclusion criteria were men or women aged over 18 years old, diagnosed with a supratentorial ischemic stroke between 24 and 72 h from onset (baseline) and with a National Institute of Health Stroke Score (NIHSS) comprised between 1 and 15. Exclusion criteria were a pre-stroke modified Rankin scale (mRS) ≥ 1, pre-stroke dementia, psychiatric disorder matching with axis 1 DSM-IV criteria, history of chronic disease compromising patient's follow-up at 1 year, and incapacity to perform the tests due to severe hemiplegia or aphasia. Demographic data and cardiovascular risk factors were recorded, as well as the treatment in the acute phase with intravenous thrombolysis. This study was part of the “Brain Before Stroke” (BBS) study, a biomedical research protocol that was accepted by the local ethical board (CPP 2012/19 2012-A00190-43). An informed consent was signed by all patients.

### Clinical evaluations

Patients were evaluated at baseline, 3 months and 1 year using a standardized cognitive and motor evaluation. This evaluation was performed in a dedicated room of the stroke unit, by a stroke neurologist together with a trained clinical research assistant, except for the Fugl-Meyer Assessment (FMA; Fugl-Meyer, 1980) which was performed by a physical therapist. Global cognitive performance was assessed by the MoCA scale (Nasreddine et al., 2005), a 30-point score including sub-scores for the evaluation of visuospatial and executive functions (5 points), naming (3 points), attention (6 points), language (3 points), abstraction (2 points), recall (5 points) and orientation (6 points). A different version of the MoCA was used at each time point to avoid learning effects. Gait speed was assessed with the 10-m walk test (10-MWT; Graham et al., 2008). Patients were asked to walk at a usual pace during two trials and the mean time to assess these two trials, expressed in seconds, was reported. No verbal instruction was given during the walking task. Global neurologic deficit and motor function were evaluated with the NIHSS at baseline, together with the total FMA and its motor sub-score (FMMA; Bushnell et al., 2015) at baseline, 3 months and 1 year. Mood changes were assessed at these three time-points using the Hospital Anxiety and Depression scale (HAD; Zigmond and Snaith, 1983). Functional outcome was evaluated using mRS at 3 months and 1 year during a medical visit (van Swieten et al., 1988). Functional independence was defined by a mRS ≤ 2.

Additionally, stroke subtypes were classified according to the Trial of Org 10172 in Acute Stroke Treatment (TOAST) classification (Adams et al., 1993). Periventricular and deep white matter hyperintensities were assessed by a stroke neurologist blinded to clinical evaluation, using the Fazekas classification (Fazekas et al., 1987) on Fluid-attenuated inversion recovery sequences (TE/TR/TI 142.8/9000/2358, FOV 24 × 24 cm^2^, matrix 288 × 224, 3 Tesla brain MRI General Electrics Medical Systems Discovery MR750W).

### Statistical analysis

Quantitative variables were expressed as means and standard deviations (SD) or medians and interquartile ratios (IQR), and qualitative variables were expressed as percentages. Comparisons of quantitative variables between baseline, 3 months and 1 year were performed using a Wilcoxon rank-sign test after verification of the required conditions.

Cognitive impairment was defined by a MoCA score <26 at 1 year post-stroke (Lees et al., 2014). A cut-off of <26 is usually used to detect single-domain cognitive impairment with a good sensitivity (Lees et al., 2014). Comparisons of demographic and clinical data between groups of MoCA <26 and ≥ 26 at 1 year were performed using unpaired two-samples Wilcoxon test or Chi 2 test for qualitative variables. Estimation of gait effects on cognitive outcome was evaluated using a generalized linear mixed model with random slopes fitted by restricted maximum likelihood (Breslow and Clayton, 1993; Chu et al., 2011). A mixed effect model has the advantage of assessing the association between changes in gait and cognitive performance over the longitudinal follow-up while including fixed effect predictors and random effects. We first performed bivariate analyses with the total MoCA score as the dependent variable, and 10-MWT, FMMA, age and gender as fixed effects. These intermediate analyses are presented as Table S1. We then performed multivariate analysis including all significant variables (*p* < 0.05) from the bivariate analysis. The model was validated by a visual inspection of histograms showing that residuals and random slopes had a nearly normal distribution. Analyses were repeated for each MoCA sub-score. Statistical analyses were performed with R software version 3.2.4, and the “lmerTest” package was used for the construction of generalized linear mixed models. Statistical significance was set at 0.05 for all tests. Statistical adjustment for multiple tests (Bonferroni correction) was used for the multivariate analysis.

## Results

Two hundred and Twelve patients were included in the analysis (71% male, mean age 64 ± SD 13 years old, 90% right-handed). Demographic data, stroke subtypes and severity of white matter hyperintensities are presented in Table 1. Clinical scores from baseline to the 1 year follow-up are summarized in Table 2. Total MoCA scores significantly improved from 24 (20–27), median (IQR) at baseline, to 26 (23–28) at 1 year (*p* < 0.001). All MoCA sub-scores improved between the three time-points. Likewise, FMMA scores improved from 96 (87–99), median (IQR) at baseline to 99 (96–100) at 1 year (*p* < 0.001). 10-MWT also improved between baseline and 1 year (11 ± 3.7 s, mean ± SD, vs. 9.9 ± 4.9, *p* < 0.001). Improvement in 10-MWT and FMMA scores mainly occurred in the first 3 months following stroke onset. There was no significant change in HAD scores between the three time-points (Table 1).

**Table 1:** **Demographic, clinical and radiological data**.

	***N* = 212**
Age, mean (*SD*)	64 ± 13
Male, *n* (%)	151 (71)
Cardiovascular risk factors, *N* (%)	
Hypertension	105 (50)
Diabetes mellitus	35 (17)
Current smoking	58 (27)
Dyslipemia	87 (41)
History of atrial fibrillation	26 (12)
NIHSS at baseline, mean (*SD*)	3.7 ± 3.3
Intravenous thrombolysis, *n* (%)	100 (47)
mRS ≤ 2, *n* (%)	
3 months	191 (90)
1 year	197 (93)
HAD, median (IQR)	
Baseline	8 (4–13)
3 months	9 (5–13)
1 year	8 (4–13)
Stroke subtypes (TOAST classification), *n* (%)	
Large-artery atherosclerosis	30 (14)
Cardioembolism	54 (26)
Small-vessel disease	20 (9)
Other	8 (4)
Undetermined	100 (47)
White matter hyperintensities (fazekas classification), *N* (%)	
Periventricular	
0	20 (9)
1	109 (52)
2	51 (24)
3	32 (15)
Deep white matter	
0	43 (20)
1	98 (46)
2	38 (18)
3	33 (16)

**Table 2:** **Clinical scores at the three time-points**.

***N* = 212**	**Baseline**	**3 months**	**1 year**	***p-*****value^†^**		
	**(a)**	**(b)**	**(c)**	**a–b**	**b–c**	**a–c**
MoCA/30, mean (*SD*)	22.1 (6.3)	24.3 (4.6)	25 (4.1)	< 0.001	< 0.001	< 0.001
Median (IQR)	24 (20–27)	25 (22–28)	26 (23–28)			
Executive and visuospatial functions/5	3.2 (1.7)	3.8 (1.2)	3.9 (1.2)	< 0.001	NS	< 0.001
Naming/3	2.8 (0.6)	2.8 (0.5)	2.9 (0.4)	NS	NS	0.03
Attention/6	4.6 (1.8)	4.9 (1.5)	5 (1.4)	0.006	NS	< 0.001
Language/3	2.2 (0.9)	2.4 (0.7)	2.4 (0.8)	0.002	NS	0.002
Abstraction/2	1.4 (0.7)	1.5 (0.6)	1.6 (0.6)	NS	0.009	0.003
Recall/5	2.4 (1.8)	3.1 (1.7)	3.3 (1.6)	< 0.001	0.03	< 0.001
Orientation/6	5.5 (1.3)	5.8 (0.7)	5.8 (0.6)	< 0.001	NS	< 0.001
10-MWT (seconds), mean (*SD*)	11.9 (3.7)	9.7 (3.9)	9.9 (4.9)	< 0.001	NS	< 0.001
**FMA, MEDIAN (IQR)**						
Total/242	227 (211–237)	236 (229–240)	237 (232–240)	< 0.001	NS	< 0.001
FMMA/100	96 (87–99)	98 (95–100)	99 (96–100)	< 0.001	NS	< 0.001

*NS, Not Significant; IQR, interquartile ratio; SD, standard deviation; 10-MWT, 10-m walk test; FMA, Fugl-Meyer Assessment; FMMA, Fugl-Meyer Motor Assessment; NIHSS, National Institute of Health Stroke Scale; mRS, modified Rankin scale; MoCA subscores are expressed as means (SD)*.

† *Wilcoxon rank-sign test*.

**Figure 1:**
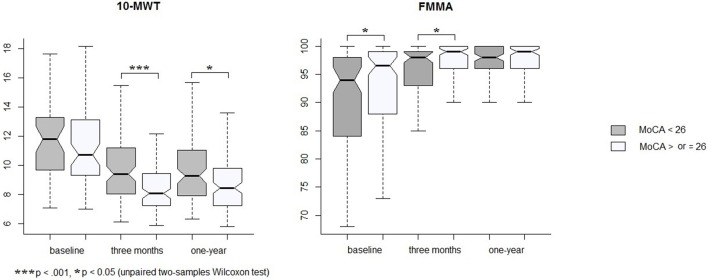
**Changes in 10-MWT and FMMA by groups of MoCA measured at 1 year**. Graphs show medians, first and third quartiles, and ranges.

At 1 year post-stroke, 99 patients (47%) were cognitively impaired with a MoCA score <26. Patients with cognitive impairment at 1 year were older (Table 3) and had 10-MWT scores significantly higher at 3 months and 1 year than patients without cognitive impairment (Figure 1). The rate of patients with functional independence at 1 year was significantly lower in the group with cognitive impairment (mRS ≤ 2: 88% vs. 97% in the group MoCA <26 and ≥ 26, respectively, *p* = 0.007). The two groups were comparable in terms of history of hypertension, diabetes mellitus, NIHSS at baseline and FMMA at 1 year.

**Table 3:** **Clinical scores by groups of MoCA measured at 1 year**.

	**MoCA <26 *N* = 99**	**MoCA ≥ 26 *N* = 113**	***p***
Age, mean (*SD*)	70 (12)	59.4 (12.6)	< 0.001^†^
Male, *n* (%)	64 (65%)	87 (77%)	0.05^‡^
Hypertension, *n* (%)	54 (54.5)	51 (45.1)	0.17^‡^
Diabetes mellitus, *n* (%)	19 (19.2)	16 (14.2)	0.3^‡^
NIHSS at baseline, mean (*SD*)	4 (3.5)	3.4 (3.1)	0.19^†^
HAD at baseline, median (IQR)	9 (3.25–14)	8 (4–11.5)	0.4^†^
HAD at 1 year, median (IQR)	9 (6–14)	8 (5–13)	0.2^†^
10-MWT at baseline, mean (*SD*)	11.7 (2.7)	12 (4.3)	0.6^†^
10-MWT at 1 year, mean (*SD*)	10.39 (4.01)	9.57 (5.63)	0.004^†^
FMMA at baseline, median (IQR)	94 (84–98)	96.5 (88.5–99)	0.03^†^
FMMA at 1 year, median (IQR)	98 (96–100)	99 (96–100)	0.2^†^
mRS ≤ 2 at 1 year, *n* (%)	87 (88)	110 (97)	0.007^‡^

*SD, standard deviation; IQR, interquartile ratio; HAD, Hospital Anxiety and Depression scale; 10-MWT, 10-m walk test; FMMA, Fugl-Meyer Motor Assessment; mRS, modified Rankin scale*.

† *Unpaired two-samples Wilcoxon test*,

‡ *Chi 2 test*.

As 10-MWT at baseline was similar in the two groups but different at 3 months and 1 year, we analyzed the relationship between changes in 10-MWT over the year of follow-up and the evolution of MoCA scores. Using a generalized linear mixed model, changes in 10-MWT were significantly associated with changes in MoCA scores (corrected *p* = 0.002, Table 4) independently of age, gender and FMMA severity. To evaluate whether the association between gait and cognition concerned a specific cognitive domain, we considered the sub-scores of the MoCA. Changes in gait performance over 1 year were associated with changes in executive functions (β = −0.04, 95% confidence interval [95% CI] −0.07 to −0.01, corrected *p* = 0.01) together with changes in recall (β = −0.06, 95% CI −0.1 to −0.03, corrected *p* = 0.002, Table 4).

**Table 4:** **Multivariate analyses**.

**Changes in scores *N* = 212**	**Estimate β**	**95% CI**	***p*^†^**
**TOTAL MoCA**			
10-MWT	−0.2	−0.24; −0.07	0.002
FMMA	−0.0006	−0.04; 0.03	NS
Age	−0.09	−0.13; −0.05	< 0.001
Male	0.05	−1.1; 1.2	NS
**EXECUTIVE AND VISUOSPATIAL FUNCTIONS**			
10-MWT	−0.04	−0.07; −0.01	0.01
FMMA	−0.003	−0.01; 0.01	NS
Age	−0.02	−0.04; −0.01	< 0.001
Male	0.3	0.04; 0.06	0.03
**NAMING**			
10-MWT	−0.002	−0.01; 0.008	NS
FMMA	0.002	−0.002; 0.007	NS
Age	−0.004	−0.008; 0.0006	NS
Male	0.009	−0.1; 0.1	NS
**ATTENTION**			
10-MWT	−0.02	−0.05; 0.01	NS
FMMA	0.007	−0.006; 0.02	NS
Age	−0.02	−0.03; −0.004	0.04
Male	0.2	−0.2; 0.5	NS
**LANGUAGE**			
10-MWT	−0.01	−0.03; 0.001	NS
FMMA	0.002	−0.005; 0.008	NS
Age	−0.004	−0.01; 0.003	NS
Male	−0.01	−0.2; 0.2	NS
**ABSTRACTION**			
10-MWT	−0.007	−0.02; 0.006	NS
FMMA	0.002	−0.004; 0.007	NS
Age	−0.008	−0.01; −0.003	0.005
Male	0.1	−0.04; 0.2	NS
**RECALL**			
10-MWT	−0.06	−0.1; −0.03	0.002
FMMA	−0.002	−0.02; 0.01	NS
Age	−0.02	−0.04; −0.009	0.005
Male	−0.3	−7; 0.1	NS
**ORIENTATION**			
10-MWT	−0.01	−0.03; 0.003	NS
FMMA	−0.009	−0.02; −0.001	NS
Age	−0.002	−0.008; 0.003	NS
Male	−0.2	−0.3; −0.002	NS

*Predictors of changes in MoCA scores and sub-scores over 1 year post-stroke considering changes in gait, adjusted for FMMA, age and sex (generalized linear mixed model)*.

NS, Not Significant; 95% CI, 95% confidence interval;

† *Corrected p-values (Bonferroni correction)*.

## Discussion

The main results of this study are that (i) in a population of mild to moderate stroke, change in gait function over 1-year post-stroke is associated with the evolution of global cognitive performance; and (ii) among the cognitive domains, executive functions and recall are the most linked to change in gait velocity.

Some cross-sectional studies have already identified a strong association between gait and post-stroke cognition but few used longitudinal evaluation to assess the interaction between these two functions (Haggard et al., 2000). In addition, these studies mostly focused on the influence of dual-task cognitive stimulation on gait patterns, which is difficult to translate into clinical practice. In order to improve the feasibility of such evaluations in routine practice, we used the MoCA which allows for an evaluation of global cognitive performance. Moreover, it offers the possibility of exploring which cognitive domains are the most relevant in tasks with cognitive interference such as gait (Nasreddine et al., 2005). The MoCA has been frequently used in studies which explored the evolution of post-stroke cognitive impairment. Recently, Delavaran et al. (2016) have reported that 61% of stroke patients with a median NIHSS at baseline of 3 had cognitive impairment 10-year post-stroke. They suggested that the MoCA was more accurate in the detection of long-term post-stroke cognitive impairment as compared to other scales such as the Mini-Mental State Evaluation. Moreover, Nijsse et al. (2017) detected 66.4 and 51.9% of post-stroke cognitive impairment defined by a MoCA score <26, after 2 months and 6 months, which is in line with the 47% of cognitive impairment reported at 1 year post-stroke in the present study. In addition, Ben Assayag et al. (2015) have recently reported that gait performance was a significant risk marker of cognitive decline 2 years after stroke.

In the current study, a strong influence of changes in gait performance on the evolution of executive functions and recall between baseline and 1 year was observed. This finding is in accordance with previous studies performed in aging and central nervous system disorders such as Alzheimer's disease, Parkinson's disease, stroke or traumatic brain injury. These studies showed an association between gait performance and impairment in attention, processing speed, verbal fluency, executive functions and memory (Al-Yahya et al., 2011). After stroke, more than two-thirds of patients were reported to suffer from working memory, executive functions and episodic memory impairment (Jaillard et al., 2010). This high frequency highlights the potentially deleterious functional impact of these forms of cognitive impairment on stroke outcome.

While still poorly understood, the close interaction between cognition and gait could be related to a cortical competition amidst cognitive and motor processes (Montero-Odasso et al., 2012). This phenomenon could be exacerbated by different brain changes which are often observed in stroke patients, the main one being the extent of white matter lesions and, as was recently described, the amyloid brain burden (Ly et al., 2012; Del Campo et al., 2016; Kim et al., 2016).

These results should be interpreted cautiously due to some limitations. First, as indicated by the low NIHSS at baseline, patients were highly selected, excluding those with severe hemiplegia or severe aphasia impeding clinical evaluation. For this reason, our results might not be generalized to more severe patients. However, the low level of physical disabilities of our sample provided the opportunity to evaluate cognition and gait without a major influence of severe neurological deficits. Second, evaluations included only the MoCA for cognitive assessment and that of gait velocity (while more discerning tests are available including dual-task paradigms). However, the observation of a strong association between the two domains through these simple tasks indicates the strength of their association and the need to evaluate these two simple markers in clinical practice. Third, although MoCA sub-scores have been used in a few studies (Lam et al., 2013; Wu et al., 2013; Washida et al., 2014), their validity for the determination of impairment in the corresponding cognitive subdomain remains to be demonstrated.

## Conclusion

This study highlights the strong association between change in gait performance and global cognitive outcome in a population of mild to moderate stroke. It reinforces the hypothesis that cognition should be evaluated in patients with gait complaints after stroke, and that post-stroke gait rehabilitation should also include cognitive rehabilitation programs.

## Ethics statement

This study was carried out in accordance with the recommendations of the French law “Jardé” relating to research involving human subjects, and the regional ethical board (CPP 2012/19 2012-A00190-43), with written informed consent for all subjects. All subjects gave written informed consent in accordance with the Declaration of Helsinki. The protocol was approved by the French Human Protection Committee of south-west and French oversea departments III.

## Author contributions

SS: acquisition of data, statistical analysis, redaction of the manuscript and revision. PR, SO, SD, MP, FR, and FM: acquisition of data and revision of the manuscript. TT: interpretation of data and critical revision of the manuscript. IS: study concept and design, statistical analysis, interpretation of data, redaction of the manuscript and revision. All authors agreed to be accountable for the content of the work.

## Funding

The study was supported by public grants from the French Agence Nationale de la Recherche within the context of the Investments for the Future Program, referenced ANR-10-LABX-57 and named “TRAIL” (Translational Research and Advanced Imaging Laboratory). The study was funded by a public grant from the French government (PHRC protocole hospitalier de recherche clinique inter-régional) funded in 2012.

### Conflict of interest statement

The authors declare that the research was conducted in the absence of any commercial or financial relationships that could be construed as a potential conflict of interest.
