# Supplementary material for: Gait Change Is Associated with Cognitive Outcome after an Acute Ischemic Stroke
Source: Front Aging Neurosci. 2017 May 18;9:153. doi: 10.3389/fnagi.2017.00153 (PMC5435741; doi:10.3389/fnagi.2017.00153)
Supplement: Supplementary file 1 [file Table1.pdf]

Supplementary material

Table S1. Bivariate analyses. Predictors of changes in MoCA scores (generalized linear mixed model)

**N = 212**	**Estimate β**	**CI 95%**	**p**
**10-MWT**	-0.19	-0.28; -0.11	< .001
**FMMA**	0.06	0.03; 0.08	< .001
**Age**	-0.11	-0.15; -0.07	< .001
**Male**	0.61	-0.59; 1.82	0.3
**Hypertension**	-1.07	-2.15; 0.02	0.06
**Diabetes mellitus**	-1.19	-2.65; 0.28	0.1

95% CI: 95% confidence interval
